# Yeast cell vacuum infusion into fungal pellets as a novel cell encapsulation methodology

**DOI:** 10.1007/s00253-023-12681-3

**Published:** 2023-07-25

**Authors:** Lara Lúquez-Caravaca, Minami Ogawa, Rewa Rai, Nitin Nitin, Juan Moreno, Teresa García-Martínez, Juan Carlos Mauricio, Juan Carlos Jiménez-Uceda, Jaime Moreno-García

**Affiliations:** 1 Department of Agricultural Chemistry, Edaphology and Microbiology, University of Córdoba, 14014 Córdoba, Spain; 2 Department of Food Science and Technology, University of California, Davis, Davis, CA 95616 USA

**Keywords:** Cell immobilization, Yeast biocapsules, Vacuum infusion, Cell retention, Winemaking

## Abstract

**Abstract:**

Immobilized yeast cells are used industrially in winemaking processes such as sparkling wine and Sherry wine production. Here, a novel approach has been explored for the infusion and immobilization of yeast cells into filamentous fungal pellets, which serve as a porous natural material. This was accomplished through vacuum application to force the yeast cells towards the core of the fungal pellets followed by culture in YPD medium to promote their growth from the interior. This method represents an improved variation of a previous approach for the assembly of “yeast biocapsules,” which entailed the co-culture of both fungal and yeast cells in the same medium. A comparison was made between both techniques in terms of biocapsule productivity, cell retention capacity, and cell biological activity through an alcoholic fermentation of a grape must. The results indicated a substantial increase in biocapsule productivity (37.40-fold), higher cell retention within the biocapsules (threefold), and reduction in cell leakage during fermentation (twofold). Although the majority of the chemical and sensory variables measured in the produced wine did not exhibit notable differences from those produced utilizing suspended yeast cells (conventional method), some differences (such as herbaceous and toasted smells, acidity, bitterness, and persistence) were perceived and wines positively evaluated by the sensory panel. As the immobilized cells remain functional and the encapsulation technique can be expanded to other microorganisms, it creates potential for additional industrial uses like biofuel, health applications, microbe encapsulation and delivery, bioremediation, and pharmacy.

**Key points:**

• *New approach improves biocapsule productivity and cell retention.*

• *Immobilized yeast remains functional in fermentation.*

• *Wine made with immobilized yeast had positive sensory differences.*

## Introduction

Yeast immobilization is defined as the physical confinement of intact yeast cells to a specific area of space (carrier) while maintaining their biological activity (Kourkoutas et al. 2004a; Lapponi et al. 2022). The immobilization of yeast cells in carriers increases ethanol tolerance and tolerance to other inhibitors, allows continuous fermentations and yeast recovery, and increases ethanol productivity, among other benefits (Kourkoutas et al. 2004a; Yuvadetkun et al. 2018; Moreno-García et al. (2018a);;. Immobilized yeast cells are currently used industrially for winemaking (i.e., sparkling wine and fino Sherry wine) and brewing. Yeast cells encapsulated in alginate beads are commercially used for the production of sparkling wine (Fumi et al. 1988; Colagrande et al. 1994; Moreno-García et al. 2018a), while yeast strains with auto-immobilization abilities, such as biofilm formation or flocculation, are used in fino Sherry production and sparkling wine and beer production and clarification, respectively (Moreno-García et al. 2018a).

Although these methods allow more flexibility to customize fermentation qualities and lower the cost of the process, their use is not widespread in many industries. Nedović et al. (2015) proposed that research should focus on low-cost systems that can be easily stored and used in a simple way in industrial volume fermenters. Furthermore, to achieve crucial factors in the production of alcoholic beverages such as consumer acceptance, safety, and/or profitability, Kourkoutas et al. (2004b) recommend that yeast carriers should be abundant in nature, cost effective, and food grade to ensure their future success in industrial application.

Yeast biocapsules are spherical yeast immobilization systems whereby yeast cells are attached to the hyphae of filamentous fungus. This system characteristics are (i) a network of active or inactive hyphae of a filamentous fungus, which can be a GRAS (generally recognized as safe) species, commonly used in the food industry and abundant in nature, and (ii) the ability to be stored for long periods in active dry format (Peinado et al. 2004a, b). These qualities can be customized depending on the needs of the application. Until now, biocapsules have been used for the production of fermentation beverages such as white wine, sparkling wine, natural sweet wine, beer, red raspberry wine, and bioethanol from molasses or starch (Peinado et al. 2005; 2006; García-Martínez et al. 2012; 2015; Puig-Pujol et al. 2013; López de Lerma et al. 2018; Ogawa et al. 2022; Liu et al. 2022).

Originally, yeast biocapsules were assembled through the co-inoculation of yeast and filamentous fungal spores and both microorganisms grew simultaneously, promoting yeast cell entrapment and attachment to the filamentous fungal pellet (García-Martínez et al. 2011; López-Menchero et al. 2021). However, this procedure restricted the microorganisms’ growth, leading to productivity limitations and low cell retention capacity (mostly during alcoholic fermentations). Furthermore, some species of yeast and filamentous fungi are not able to co-culture in the same medium or do not attach to one another, thus restricting the range of fungal species/strains combinations. To overcome these limitations, a new methodology to assemble biocapsules has been proposed. This technique involves four steps: (i) culture filamentous fungal spores and yeast cells separately to avoid microorganism competition and promote biomass production, (ii) mix resulting pellets and yeasts suspension, and yeast cell infusion into the pellets via vacuum to increase yeast cell population in the pellet core, (iii) culture in YPD liquid medium to promote further attachment of yeast and pellet, and (iv) wash to remove non-attached cells in the pellet surface. The biocapsules that resulted from the new methodology have been named “microbial biocapsules.”

In this study, we examine the performance of biocapsules assembled by mixing yeast pellets and vacuum infusion in an alcoholic fermentation of grape must. Cell immobilization yields, yeast cell leakage after fermentation, fermentation yields, chemical profile, and sensorial features of the produced wine have been analyzed and compared with fermentations conducted with biocapsules made by co-inoculation of yeast and filamentous fungal spores, and free yeast cells as the conventional method used in the wine industry.

## Materials and methods

### Microorganisms and growth media

*Saccharomyces cerevisiae* G1 strain (ATCC: MYA-2451; University of Córdoba Collection, Córdoba, Spain), a biofilm-forming yeast used in the biological aging of Sherry-type wines, and the filamentous fungus (ff) *Aspergillus oryzae* 76–2 (FST 76–2; UC Davis Phaff Culture Collection, Davis, CA, USA) were used in this study. YPD (in g/L: yeast extract, 10; peptone, 20; dextrose, 20) medium was used as a yeast pre-culture medium to grow yeasts overnight at 175 rpm, 28 °C (Moreno-García et al. 2018b). The ff was cultured on a sporulation medium (g/L): corn meal agar, 17; yeast extract, 1; glucose, 2; agar, 20; for 7 days at 28 °C.

### Yeast immobilization procedures

Yeast cells were immobilized in two different formats: yeast biocapsules assembled by the co-inoculation technique (COYB) and yeast biocapsules assembled by mixing yeast pellets followed by vacuum infusion technique (MVYB). Free yeast cells (FY) were used as the control. COYB were produced in a biocapsule formation medium (BFM) composed of (g/L): yeast nitrogen base medium without amino acids (Difco™), 6.7; gluconic acid, 5; and buffered to pH 7 with Na_2_HPO_4_, 7.2; and KH_2_PO_4_, 3.6. In a sterile 250-mL Erlenmeyer flasks containing 150 mL BFM, 3 × 10^4^ *A. oryzae* spores/mL and 1 × 10^6^ yeast cells/mL were co-inoculated and shaken at 175 rpm, 28 °C, for 7 days (Moreno-García et al. 2018c; Ogawa et al. 2020). ff was inactivated by submerging COYB into a high-sugar medium (YP + 250 g/L dextrose) for 12 days (García-Martínez et al. 2011). To produce MVYB, separate cultures of yeasts and ff pellets were prepared before the yeast vacuum infusion. Yeast cells were cultured in YPD overnight at 175 rpm, 28 °C (as previously indicated). ff spores were harvested from the sporulation agar medium into a vessel with sterile DI (deionized water) water, vortexed and sonicated for 5 min to avoid agglomeration and inaccuracy in the inoculation. A controlled spore population was inoculated to reach a final population of 1 × 10^6^ spores/mL in a fungal pellet culture medium (FPM) consisting of (g/L): glucose, 60; yeast extract, 3; NaNO_3_, 3; K_2_HPO_4_, 1; MgSO_4_, 0.5; KCl 0.5; and FeSO_4_; 0.01 and buffered to pH 5.5 with HCl. ff spores were cultured in FPM for 3 days at 175 rpm, 30 °C; to form the fungal pellets. Fungal pellets were inactivated by autoclave (1 atm overpressure, 20 min, 121 °C).

Cultured yeast cells and ff pellets were collected, and a proportion of 1:1 wet weight yeast:ff pellet was immersed in a 50-mL Falcon tube with sterile DI water. This suspension was subjected to vacuum infusion (< 0.3 atm pressure) for 1 min using a Bonsenkitchen system (Oakwood, GA, USA), forcing the microbial cells inside the tight hyphae matrix of the ff pellets. To confirm infusion, OD_580_ was measured in the cell suspension both before and after the vacuum step; a 20% reduction in OD_580_ was obtained. Yeast cell-infused ff pellets were submerged into a YPD medium and cultured overnight at 175 rpm, 28 °C. Finally, the obtained MVYB were rinsed with sterile DI water, to remove the cells located in the surface, considering that loose cells could lead to cell leakage during the subsequent fermentation. The MVYB assembling methodology has been submitted to patent application (Application number: 64/411,843).

### Fermentation conditions

A fermentation in grape must (GM) was carried out. The must was 17.75 Brixº, 50 mg/L SO_2_, 4.26 ± 0.01 pH, 0.25 ± 0.02 volatile acidity (expressed as grams of acetic acid/L), and 12.25 ± 0.85 titratable acidity (expressed as grams of tartaric acid/L) obtained from grapes of the Pedro Ximénez variety in the Montilla-Moriles winemaking region (Córdoba, South Spain). Previous to fermentation, GM was subjected to centrifugation (7000 rpm, 15´; Beckman Coulter J2-HS Centrifuge, ø 30 cm), and further filtration (Supor® 450 Membrane Disc Filters, 0.45 µm—142 mm, tabbed (25/pkg) and Supor® 450 Membrane Disc Filters, 0.45 µm—142 mm, tabbed (25/pkg) both from Cytiva, Marlborough, MA, USA) to remove solid particles and potential contaminants. The GM fermentations were carried out in 1-L Erlenmeyer flasks containing 500 mL of must at 21 °C for 17 days or until fermentation was complete (residual sugars < 1 g/L). All fermentations (MVYB, COYB, and FY) were run parallelly and monitored via weight loss of flask due to the CO_2_ released during fermentation (Bezenger 1985). All media were inoculated with a population of 1 × 10^6^ yeast cells/mL or the equivalent wet weight (WW) in case of immobilization formats. Yeast cells in MVYB and COYB were subjected to a cell-carrier detachment step prior to cell counting and inoculation: five random weighted biocapsules per culture flask were submerged in a solution of 0.1 M NaCl, disrupted with a tissue grinder (Kisker Biotech, Steinfurt, Germany) and sonicated for 20 min until a homogeneous suspension was obtained. Using this technique, yeast cells and *A. oryzae* hyphae segments were mixed together, and under a 40 × objective microscope, yeast cells and the hyphal debris could be easily differentiated.

### Yeast immobilization yields and general enological analysis

Non-immobilized and immobilized yeast populations were determined by a cell-carrier separation step when required (previously described) and cells counted with a Thoma chamber and a 40 × objective on a light microscope. Furthermore, MVYB were imaged using a scanning electron microscope (SEM) to visualize the yeast cells inside the pellets following the protocol described in García-Martínez et al. (2012). The resulting samples were examined and photographed with a Thermo Fisher Quattro S Environmental SEM (Waltham, MA, USA).

Parameters commonly measured for wine (ethanol, total and volatile acidity, and pH) were quantified using methods recommended by the International Organization of Vine and Wine. Glucose and fructose concentrations were quantified using the D-Fructose/D-Glucose assay kit (ref: 10,139,106,035, R-Biofarm, Darmstadt, Hessen, Germany).

### Major aroma compounds and polyols quantification

A gas chromatograph (GC) Agilent 6890 (Palo Alto, CA, USA) equipped with a fused silica capillary column (60 m, 0.25 mm diameter, 0.4 m film) connected to a flame ionization detector (FID) was used to measure the major wine volatile aroma compounds and polyols that contribute to the organoleptic properties. Chromatographic parameters were established using the Peinado et al. (2004a, b) methodology. The concentration of the wine compounds was obtained by direct injection in the GC inlet of 1 µL mixture that consisted of 1 mL of an internal standard solution (1 g/L of 4-methyl-2-pentanol in 14% (v/v) ethanol) and 10 mL of wine. The oven initial temperature was 45 °C for 15 min, ramped up to 190 °C at 4 °C each min, and maintained for 35 min. The injector and detector were set to 270 °C and 300 °C, respectively. The carrier gas was helium, and split mode injections were carried out (1:10). The flow rate was programmed as follows: 0.7 mL/min for 16 min, ramped up to 1.1 mL/min at 0.2 m/min for 52 min. To identify specific chemicals based on their mass spectra, purified substances from Thermo Fisher Scientific (Waltham, MA, USA), Merck (Darmstadt, Germany), and Sigma-Aldrich (St. Louis, MO, USA) were added to the chromatographic peaks, and quantification was carried out using a calibration table built from standard solutions with known concentrations (Vararu et al. 2016). The measured chemicals were identified and validated by GC–MS (gas chromatography-mass spetrometry) using the same capillary column, temperature, and helium protocols on an Agilent 7890 A with MSD-5975-C (Wilmington, DE, USA).

### Sensory analyses

To validate the MVYB application for winemaking, blind sensory analysis was carried out in MVYB, COYB, and FY wines by a tasting panel of 9 judges, all expert tasters. Randomized samples of 25–30 mL were served at 8–10 °C in clean and clear glasses with random letter labeling. The panel scored the wine samples visually, aromatically, and gustatorially on a scale of 0 to 10, with 10 representing the highest level of intensity and 0 signifying absence or low intensity. To determine if the panel could tell the difference between wine produced using the traditional way (FY) and the novel proposed method (MVYB), two distinct triangular tasting sessions were conducted in accordance with Puig et al. (2013). Three series of three glasses each were reviewed for each tasting session. Each series consisted of three samples: two identical (using the same method for yeast inoculation) and one different. In order to distinguish between wine made with MVYB and FY, the taster was asked to identify the sample that was different.

### Statistical analysis

Statgraphics v. XVI.I software (StatPoint Technologies Inc., Warrenton, VA, USA) was used for multiple variable analysis (MVA) principal component analysis (PCA) and multiple sample comparisons by homogeneous groups (HG), to determine significant differences between the various immobilization conditions.

## Results

### Yeast cell immobilization

**Fig. 1:**
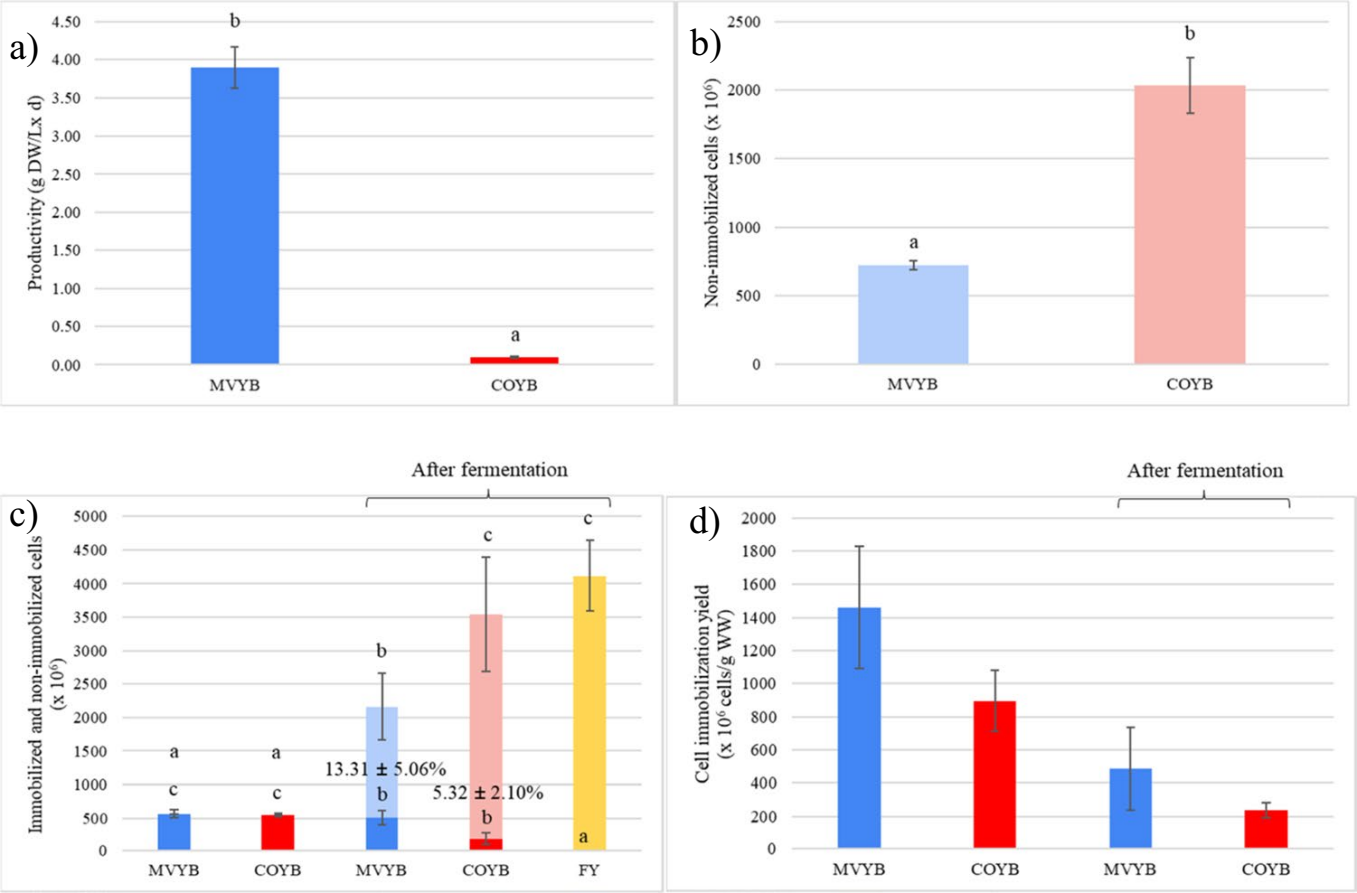
**a** Biocapsule productivity in gram dry weight per liter of culture medium and cell immobilization parameters in yeast biocapsules assembled by mixing yeast pellets followed by vacuum infusion technique (MVYB), by the co-inoculation technique (COYB), and free yeasts (FY) before and after fermentation of the grape must (GM): **b** total non-immobilized cells before fermentation after the biocapsule assembling procedures, **c** total immobilized and non-immobilized cells before and after fermentation, **d** cell immobilization yield per gram of biocapsule in wet weight before and after fermentation. Darker color bars represent yeasts immobilized, and lighter color bars represent non-immobilized or free yeasts. The error bars represent the standard deviation over averages from biological replicates (*n* = 3). The letters above graphics represent the homogeneous groups statistically significantly differing in parameters among the conditions; in **c**, homogeneous groups on top represent non-immobilized cells while those in the button refer to immobilized cells

Biocapsule productivity by using the two methods (mixing yeast pellets followed by vacuum infusion technique and by fungal spore and yeast co-inoculation) and cell immobilization parameters quantified in the biocapsules assembled before and after alcoholic fermentation are shown in Fig. 1. Figure 1a shows a notable increase in productivity (dry weight of biocapsules produced per liter of medium and day) up to 37.40-fold when using the mixing yeast pellets followed by vacuum infusion technique. Total non-immobilized cells that remain after the biocapsule assembly were more abundant in COYB versus MVYB (2033.33 ± 202.07 × 10^6^ cells and 720.00 ± 32.45 × 10^6^ cells, respectively) while cell immobilization yields were found to be higher in MVYB than in COYB (1461.85 ± 368.05 cells/g WW and 895.24 ± 183.69 × 10^6^ cells/g WW, respectively or 40.43 ± 6.40% and 19.37 ± 2.17% of the total yeast cell population, respectively). The same parameters were measured after GM fermentation. Lower cell leakage and growth were observed in MVYB fermentations compared to COYB (1650 ± 500 × 10^6^ non-immobilized cells and 3350 ± 850 × 10^6^ non-immobilized cells, respectively) (Fig. 1b); both results were below the FY condition (4116.67 ± 850 × 10^6^ cells) signifying that both immobilization systems can ease wine clarification processes. For the total immobilized cells after fermentation (Fig. 1c), MVYB immobilized a total of 510.76 ± 104.36 × 10^6^ cells (13.31 ± 5.06% of the total population) whereas COYB 190.33 ± 88.67 × 10^6^ cells (5.32 ± 2.10%). Cell immobilization yield per gram of wet weight was also higher in MVYB (487.10 ± 175.44 × 10^6^ cells/g WW versus 238.45 ± 45.78 × 10^6^ cells/g WW in COYB).

**Fig. 2:**
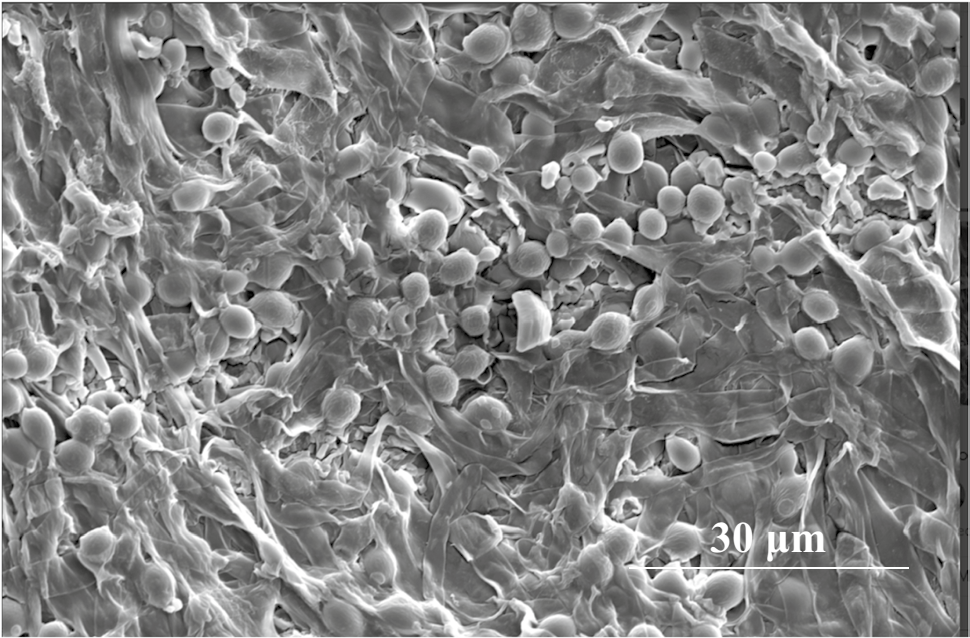
Immobilized yeast cells inside fungal pellets in biocapsules made using the vacuum infusion technique in a scanning electron microscope (SEM) image

Figure 2 shows a SEM image wherein it can be observed yeast cells that are immobilized inside the filamentous fungus pellet in biocapsules assembled by mixing yeast pellets followed by vacuum infusion technique (MVYB).

### Alcoholic fermentation rates

**Fig. 3:**
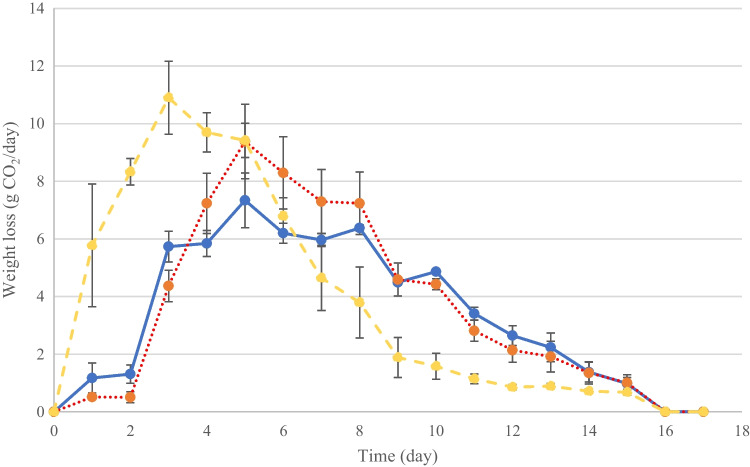
Fermentation kinetics found by the evolution of CO_2_ production and release during the fermentation of grape must (GM) by biocapsules assembled by mixing yeast pellets followed by vacuum infusion technique (MVYB) in blue line, by the co-inoculation technique (COYB) in red dots, and by free yeast cells (FY) in yellow dashed line. The error bars represent the standard deviation over averages from biological replicates (*n* = 3)

Figure 3 shows the mass loss evolution due to CO_2_ release during the GM fermentation process. The highest fermentation rates were reported on day 3 in the FY fermentation (10.90 ± 1.27 g CO_2_/day). In MVYB and COYB fermentations, the mass loss peaks were reported at day 5 with 7.34 ± 0.95 g CO_2_/day; and 9.38 ± 1.29 g CO_2_/day, respectively.

### General enological parameters

**Fig. 4:**
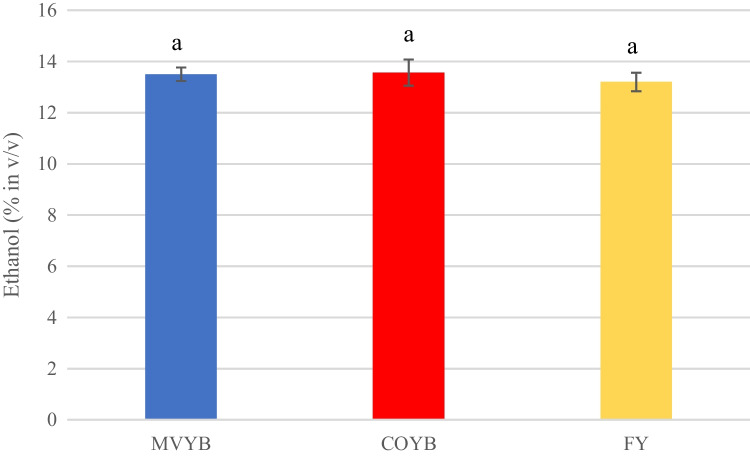
Ethanol concentration at the end of the fermentation of grape must (GM) produced with biocapsules assembled by mixing yeast pellets followed by vacuum infusion technique (MVYB) in blue, by the co-inoculation technique (COYB) in red, and by free yeast cells (FY) in yellow. The error bars represent the standard deviation over averages from biological replicates (*n* = 3). The letters above bars represent the significantly different homogenous groups in parameters among the inoculum conditions

Ethanol concentration of wine produced with different inoculation formats is shown in Fig. 4. Similar values were obtained in all tested conditions. Although no significant differences were reported, the highest values were detected in COYB fermentation (13.57 ± 0.51% in v/v), followed by the MVYB (13.50 ± 0.26% in v/v) and FY (13.20 ± 0.36% in v/v).

A pH decrease in GM (4.26) occurred as a consequence of the metabolic activity of the yeasts (Table 1). There was a higher reduction in fermentations utilizing immobilized yeasts. As for the titratable or total acidity, values did not fluctuate among the different studied conditions. While COYB and FY wines showed similar values (~ 3.90 g H_2_T/L or grams of tartaric acid equivalents per liter), the highest value was obtained in MVYB with 4.26 ± 0.07 g H_2_T/L. Volatile and fixed acidities differed more between wines: FY wine had the highest volatile acidity with 0.88 ± 0.05 g ACH/L or grams of acetic acid equivalents while the lowest was reported in the COYB with 0.45 ± 0.07 g ACH /L; all below 1.2 g ACH/L, which could mean that wine has been acidified by the action of acetic acid bacteria that negatively influences on the wine organoleptic properties. Highest value for fixed acidity was reported in MVYB wine (3.50 ± 0.07 g ACH/L), while the lowest was in FY (3.04 ± 0.16 g ACH/L). COYB wines obtained similar values to MVYB (3.40 ± 0.09 g ACH/L).

**Table 1:** Acidity and pH parameters obtained from wines produced with biocapsules assembled by mixing yeast pellets followed by the co-inoculation technique (COYB), vacuum infusion technique (MVYB), and free yeast cells (FY)

	COYB	MVYB	FY
pH	3.77 ± 0.01^a^	3.75 ± 0.02^a^	3.90 ± 0.02^a^
Titratable acidity (g H_2_T /L)	3.85 ± 0.0^4a^	4.26 ± 0.07^b^	3.92 ± 0.11^a^
Volatile acidity (g ACH/L)	0.45 ± 0.07^a^	0.76 ± 0.06^b^	0.88 ± 0.05^b^
Fixed acidity (g ACH/L)	3.40 ± 0.09^b^	3.50 ± 0.07^b^	3.04 ± 0.16^a^

Letters represent homogenous groups which differed statistically significant in parameters among the strains

### Major concentration of aromatic compounds and polyols

Fifteen different compounds were measured by gas chromatography (Table 2). These compounds are by-products of the GM fermentation. Among them, eight did not overpass the odor threshold (OT), meaning that they did not contribute noticeable organoleptic properties to the wine. On the other hand, isobutanol, 2-phenylethanol, acetaldehyde, acetoin, ethyl acetate, ethyl lactate, and 2,3-butanediol (levo) were quantified over their OT in all or some of the wines produced.

**Table 2:** Major volatile compounds in wines produced with biocapsules assembled by mixing yeast pellets followed by the vacuum infusion technique (MVYB), the co-inoculation technique (COYB), and free yeast cells (FY)

Major volatile compounds		CAS	OT (mg/L)	Odor/flavor description	MVYB	COYB	FY
Alcohols	Methanol (mg/L)	67–56-1	668	Chemical, medicinal	26.53 ± 0.12^a^	31.09 ± 2.21^a^	54.21 ± 2.57^a^
	1-Propanol (mg/L)	71–23-8	830	Ripe fruit, alcohol	50.97 ± 0.76^a^	77.29 ± 6.83^b^	45.39 ± 1.46^a^
	Isobutanol (mg/L)	78–83-1	40	Alcohol, wine like, nail polish	144.56 ± 6.61^b^	139.08 ± 19.18^b^	86.84 ± 16.01^a^
	2-Methyl-1-butanol (mg/L)	137–32-6	NF	Cooked roasted aroma with fruity or alcoholic undertones	30.28 ± 0.48^b^	36.16 ± 2.12^c^	26.87 ± 1.1^a^
	3-Methyl-1-butanol (ISO) (mg/L)	125–51-3	NF	Disagreeable	233.69 ± 6.62^b^	192.6 ± 18.45^a^	173.07 ± 17.37^a^
	2-Phenylethanol (mg/L)	60–12-8	10	N.f	30.4 ± 1.89^ab^	37.36 ± 8.6^b^	22.59 ± 2.33^a^
Acetaldehyde and derivatives	Acetaldehyde (mg/L)	75–07-0	10	Over-ripe apple	181.19 ± 11.06^a^	211.43 ± 29.36^a^	250.09 ± 51.46^a^
	Acetoin (mg/L)	53,584–56-8	30	Buttery, cream	93.97 ± 9.14^a^	100.22 ± 13.82^a^	101.96 ± 6.00^a^
	1,1-Diethoxyethane (mg/L)	105–57-7	NF	Refreshing, pleasant, fruity-green	-	1.44 ± 0.3^b^	1.96 ± 0.21^ab^
Esters	Ethyl acetate (mg/L)	141–78-6	7.5	Pineapple, varnish, balsamic	46.18 ± 0.5^a^	41.29 ± 7.45^a^	43.29 ± 6.36^a^
	Ethyl lactate (mg/L)	97–64-3	7.5	Strawberry, raspberry, buttery	10.17 ± 0.17^b^	9.91 ± 0.61^b^	-
Polyols	Diethyl succinate (mg/L)	123–25-1	100	Over-ripe, lavender	3.82 ± 0.91^a^	7.05 ± 0.55^b^	3.41 ± 0.53^a^
	2,3-Butanediol (levo) (mg/L)	24,347–58-8	668	Buttery, creamy	860.95 ± 79.19^a^	678.21 ± 107.29^a^	1212.72 ± 161.96^b^
	2,3-Butanediol (meso) (mg/L)	5341–95-7	668	Buttery, creamy	315.34 ± 27.65^a^	349.63 ± 36.81^a^	389.18 ± 49.01^a^
	Glycerol (g/L)	56–81-5	NF	N.f	10.19 ± 1.38^b^	7.41 ± 1.31^a^	10.23 ± 1.17^b^

This table also provides the CAS number for the different compounds, odor threshold (OT), and odor/flavor descriptions. Cells shadowed represent those compound concentrations above the compound odor threshold. NF refers to values not found in the literature. Letters represent homogenous groups which differed statistically significantly in parameters among the strains. Threshold values were taken from Zea et al. (2007) and Moreno-García et al. (2015)

**Fig. 5:**
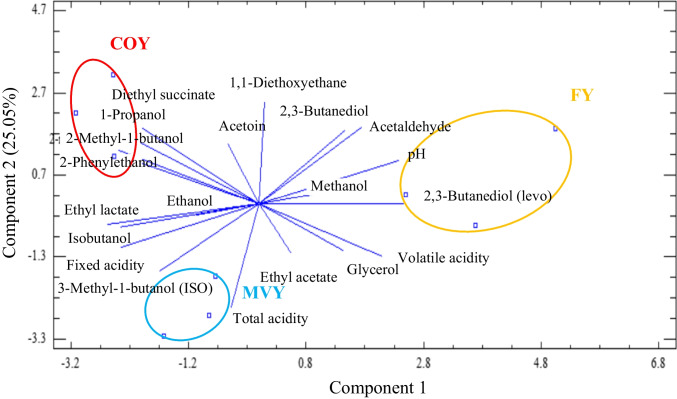
Principal component analysis (PCA) stating the contribution of different chemical parameters for wines when fermenting grape must (GM) with biocapsules assembled by mixing yeast pellets followed by vacuum infusion technique (MVYB) in blue, by the co-inoculation technique (COYB) in red, and by free yeast cells (FY) in yellow

These metabolites and general chemical parameters were also subjected to a PCA as shown in Fig. 5 to determine compounds which contributed to differentiate between conditions (dark blue lines). Components 1 (44.27%) and 2 (25.05%) explain 69.32% of the variance. Ethanol, ethyl lactate, isobutanol, fixed acidity, 3-methyl-1-butanol, total acidity, ethyl acetate, glycerol, and volatile acidity have negative projections over the rest of the metabolites in component 1, while acetoin, diethyl succinate, 1-propanol, 2-methyl-1-butanol, 2-phenylethanol, ethanol, ethyl lactate, isobutanol, fixed acidity, 3-methyl-1-butanol (ISO), and total acidity have negative projections over the rest of the metabolites in component 2. In this PCA, all conditions were clearly separated; COYB replicates are positioned to the top left, MVYB are found on the bottom left and FY replicates are found on the right with two positive and one negative projections on component 2.

### Sensory analysis

**Fig. 6:**
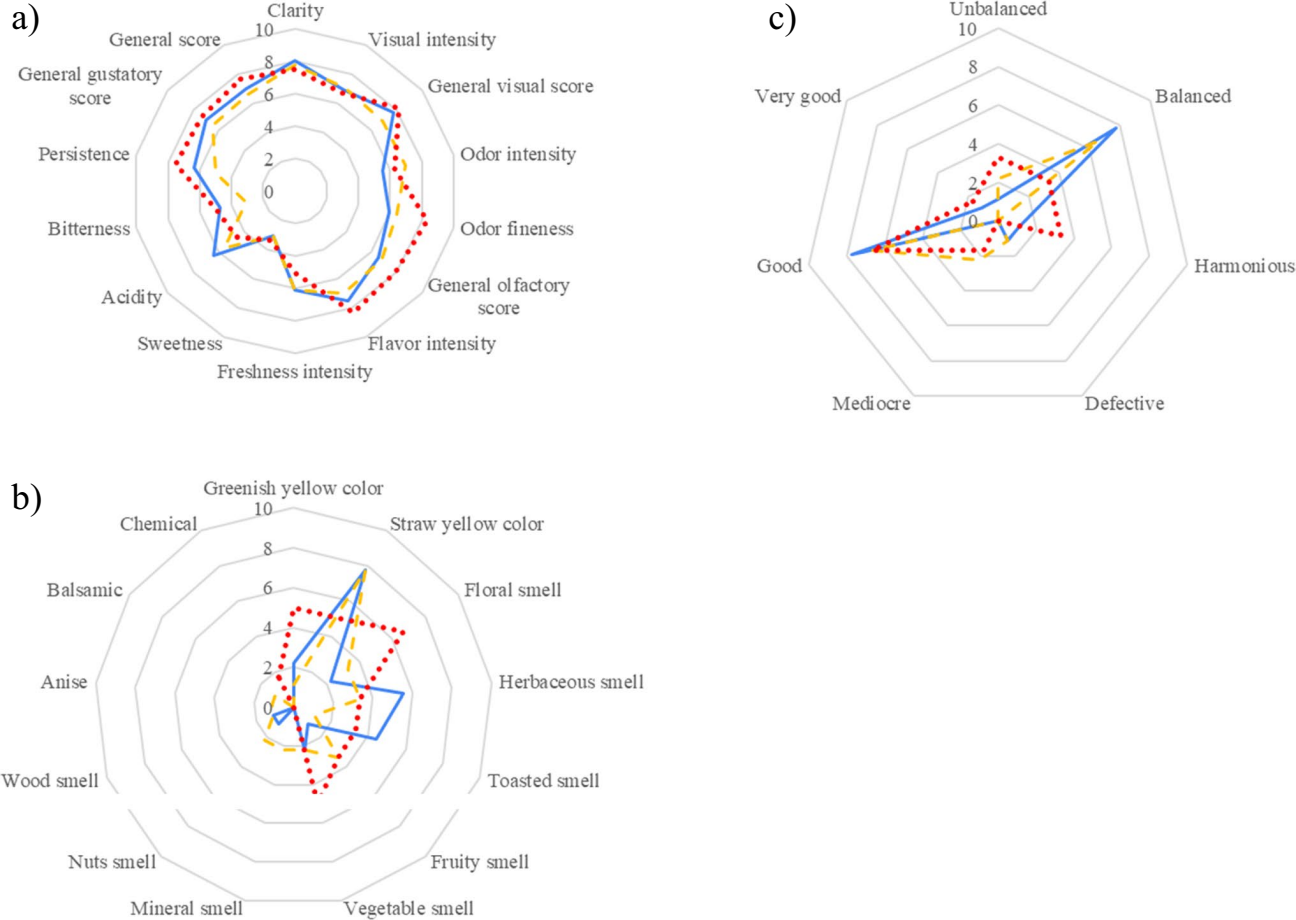
Sensory profile plot in wines produced with biocapsules assembled by mixing yeast pellets followed by vacuum infusion technique (MVYB) in blue lines, by the co-inoculation technique (COYB) in red dots, and by free yeast (FY) in yellow dashed lines. Values represent averages from evaluation scores (*n* = 9). Specific attributes are represented in **a** and **b**, while general scores are represented in **c**

Of judges, 27.78% could distinguish between MVYB and FY wines. This value is below 55% which is the minimum to consider a significant difference level between two samples according to ISO 4120 (2004). Similar organoleptic profiles were reported for MVYB, COYB, and FY wines; however, more panelists detected a toasted aroma with significantly higher odor intensities and fineness in wines produced with MVYB (Fig. 6). Compared to FY wines, MVYB wines exhibit higher levels of acidity, bitterness, persistence, and aromas reminiscent of herbs or toasting. On the other hand, they have a lower intensity of odor and exhibit floral, fruity, mineral, and nutty notes. Despite no significant chemical differences, MVYB wines outperformed traditional FY wines in both visual and gustatory categories overall.

## Discussion

### Mixing yeast pellets followed by vacuum infusion technique improves biocapsule productivity, cell immobilization, and prevents cell leakage in alcoholic fermentation

Cell leakage is a typical issue in immobilization technologies (Moreno-García et al. 2018a; Lapponi et al. 2022). To understand and resolve the issue, research efforts are geared towards improving cell confinement while maintaining bioactivity and productivity (Nedovic and Willaert 2006; Moreno-García et al. 2018b, c; Ogawa et al. 2019; López-Menchero et al. 2021). In this study, we attempted to lower the cell detachment during alcoholic fermentation by using biocapsules that were assembled with a novel method based on the yeast cell vacuum infusion into fungal pellets. As noted in this study, fewer cells remain unattached to the carrier during this immobilization process (see Fig. 1b) and consequently, a greater number of cells are trapped per gram of biocapsules (Fig. d), thus, demonstrating a higher potential versus COYB for inoculation purposes (less grams of biocapsules required to start the fermentation). More importantly, mixing yeast pellets followed by vacuum infusion technique decreased cell leakage during alcoholic fermentation by twofold (Fig. 1c) while maintaining a higher cell population entrapped/attached to the fungal carriers (Fig. 1c and d). In comparison with alcoholic fermentations carried out by FY, MVYB lowered the yeast cell population in suspension by 2.5-fold; this is advantageous for wine clarification by precipitation or filtration. One possible explanation for these findings is that the vacuum infusion step facilitates yeast cells’ access to areas within the hypha structure that would otherwise be inaccessible (i.e., if using the CMYB technique). The carrier may leak yeast cells that are fixed on the pellet surface. In MVYB, yeast cells continue to proliferate in the core of the filamentous fungal pellet when they are submerged in YPD culture, leading to higher immobilization yields.

From our literature search, we did not find any other works in which vacuum infusion is used to entrap microbial cells in porous matrices. However, there are studies that evaluated chemicals (i.e., bioactive compounds such as curcumin and fisetin) or enzymes vacuum infusion (i.e., pectinmethylesterase) into microbial cells (i.e., yeast cells), fungal cell wall particles, or plant cells (Banjongsinsiri et al. 2004; Guillemin et al. 2008; Young et al. 2017; Wu et al. 2023). Young et al. (2017) reported a 2- and threefold increase of fisetin and curcumin into yeast microcarriers, respectively, compared to the diffusion-limited methodologies, whereas Banjongsinsiri et al. (2004), Guillemin et al. (2008), Young et al. (2017), and Wu et al. (2023) reported enhanced firmness in plants and integrity among cells compared to controls (fresh non-infused and water-infused control).

López-Menchero et al. (2021) coated yeast biocapsules with a 0.2% (w/v) alginate layer, enhancing immobilization yield by sixfold after a GM fermentation, which is higher than what was attained in this study (Fig. 1). However, it should be noted that these assays were conducted using the co-inoculation technique that presents other drawbacks versus mixing yeast pellets followed by vacuum infusion technique: significantly lower biocapsule productivity (Fig. 1a) and higher contamination risk due to the usage of gluconic acid as the sole carbon source when assembling CMYB—a carbon source difficult to assimilate by yeast (Ogawa et al. 2020) and easy to assimilate by opportunistic microorganisms like filamentous fungi. MVYB also overcomes the limitation of immobilizing only yeast cells as other microorganisms, such as bacteria or microalgae (data included in patent application). The reason for this is that the entrapment and immobilization of cells relies on a physical process (such as vacuum) rather than a biological one (such as the ability of cells to grow and cohere in the presence of the filamentous fungus). Nonetheless, to further increase cell loading and improve cell retention in fungal pellets, further techniques should be considered such as multiple vacuum-facilitated infusion rounds or biocapsule coating with alginate or other biopolymers (e.g., cellulose acetate, chitosan, pectin and gelatin, collagen).

### Yeasts in MVYB are functional and do not significantly affect the chemical and organoleptic profile of wines

In order to test the functionality, or bioactivity, of yeast cells vacuum-infused in fungal pellets, three parallel alcoholic fermentations of GM were conducted: inoculating with CMYB, MVYB, and FY. Fermentation kinetics differed between conditions, but the final concentration of ethanol (Fig. 4) and most of the alcoholic fermentation by-products were similar to those produced in FY condition. Figure 3 represents the amount of CO_2_ produced and released over time which follows a sigmoid curve, typical of the equation of fermentation found by Gompertz and Lineweaver–Burk (Callone et al. 2008; López-Menchero et al. 2021). This figure clearly shows a difference in FY control condition. Immobilization-induced changes in cell development, physiology, and metabolism, and/or cell surface constraints may explain lower CO_2_ releases (10.90 CO_2_/day, 9.38 CO_2_/day, and 7.34 CO_2_/day in FY, COYB, and MVYB fermentations; respectively) and later fermentation peaks (at day 5) when using immobilized yeasts (Peinado et al. 2006; Sroka et al. 2017; Moreno-García et al. 2018a). COYB curve is more similar to FY, possibly due to a higher yeast cell leakage. However, several authors observed when immobilized yeast cells are re-used, growth lag phase shortens and fermentation rates equal or even exceed those produced by free yeast cells (García-Martínez et al. 2012, 2015; Puig-Pujol et al. 2013).

After all residual sugars were consumed, and the wines obtained were subjected to a chemical characterization. We observed that isobutanol, 2-phenylethanol, acetaldehyde, acetoin, ethyl acetate, and 2,3-butanediol (levo) were quantified above their OT in all studied conditions (wines made with MVYB, COYB, and FY) (Table 2). Only ethyl lactate was detected over its OT in the immobilized formats and not in FY. This compound provides pineapple, varnish, and balsamic aromas (Zea et al. 2007; Moreno-García et al. 2015). Regarding the alcohols, two compounds were present in a high amount in the cells immobilized in biocapsules versus free yeasts: isobutanol and 2-phenylethanol. The first offers odors of alcohol or nail polish, and higher concentrations were found in similar studies about the characteristics of yeast biocapsule’s fermentations (Peinado et al. 2004a, b; García-Martínez et al. 2015). 2-Phenylethanol is often desired due to its “rose” or “floral” aroma and is rarely found in wild-type strains of *S. cerevisiae* (Cordente et al. 2018).

Acetaldehyde and acetoin are also very important volatile compounds perceived in wine (Liu and Pilone 2000). Acetaldehyde offers a fruity aroma when above 125 mg/L and acetoin offers creamy aromas. These two compounds were detected in similar quantities in all tested conditions. On the other hand, yeast cells in immobilized formats decreased in 2,3-butanediol (levo), although still over its OT. This compound also provides buttery and creamy aromas.

Additionally, an organoleptic analysis was carried out. The tasting panel could not discriminate between MVYB and FY wines (only 27.78% of the judges were able to do so) and the analysis resulted in slightly higher overall ratings for the biocapsule-produced wines probably due to a higher toasted smells, acidity, bitterness, and persistence. These organoleptic differences could be correlated to the differences in concentrations of those volatiles that overpassed their odor threshold (isobutanol, ethyl lactate, 2,3 butanediol (levo), and 2-phenylethanol) and the production of unanalyzed metabolites (such as minor volatile compounds) (Martínez-García et al. 2021).

Given that the use of yeast immobilization systems in industrial settings offers several advantages over free yeasts, as previously discussed, the finding that MVYB and FY wines display overall identical chemical and organoleptic profiles (and even superior ratings) is a promising result.

Here, a new methodology to immobilize yeast cells into fungal pellets, yeast and fungal pellet mixing with vacuum infusion, has been compared with a yeast cell-fungal spores co-culture technique. From our understanding, this is the first time that vacuum is used to infuse whole cells in a porous matrix. Results showed that the mixing-infusion technique significantly increases biocapsule productivity (37.40-fold), prevents cell leakage up to two-fold during GM fermentation, and entraps more cells (almost three-fold). These results are promising to improve media clarification and cell reutilization in industrial settings which are of high relevance for the wine or beer sectors by using a natural carrier such as an inactive pellet of a GRAS fungus (*A. oryzae*) widely used for food purposes. After a chemical and sensory analysis of the produced wines, it was reported that yeasts immobilized with the novel method are biologically functional and that most of the parameters quantified did not show significant differences chemically but showed organoleptical improvement when compared to wines made with the conventional method (suspended yeast cells). This study demonstrates that vacuum infusion is efficient for microbial cell (i.e., yeast cells) entrapment into porous matrices (i.e., fungal pellets) while maintaining bioactivity of the immobilized cells. These results suggest that this method could potentially be extended to include other types of microorganisms and uses such as biofuel, health applications (e.g., immobilizing probiotics in edible fungi), encapsulating and delivering microbes (e.g., beneficial plant bacteria for biocontrol or nitrogen-fixing bacteria), bioremediation, and pharmacy (e.g., immobilizing antibiotics, hormones, or drug-producing cells for synthesizing pharmaceutical drugs and increasing production through synergy with a specific type of filamentous fungus).

## Data Availability

All data generated or analyzed during this study are included in this published article.

## Declarations

### Patents

The microbial biocapsule assembling methodology has been submitted to patent application (Application number: 64/411,843).
